# ChatGPT in medical school: how successful is AI in progress testing?

**DOI:** 10.1080/10872981.2023.2220920

**Published:** 2023-06-12

**Authors:** Hendrik Friederichs, Wolf Jonas Friederichs, Maren März

**Affiliations:** a Medical School OWL, Bielefeld University, Bielefeld, Germany; b Faculty of Mechanical Engineering, RWTH Aachen University, Aachen, Germany; c Charité– Universitätsmedizin Berlin, Kooperationspartner der Freien Universität Berlin, Humboldt-Universität Zu Berlin, Progress Test Medizin, Charitéplatz 1, Berlin, Germany

**Keywords:** Medical education, progress test, learning, artificial intelligence, machine learning

## Abstract

**Background:**

As generative artificial intelligence (AI), ChatGPT provides easy access to a wide range of information, including factual knowledge in the field of medicine. Given that knowledge acquisition is a basic determinant of physicians’ performance, teaching and testing different levels of medical knowledge is a central task of medical schools. To measure the factual knowledge level of the ChatGPT responses, we compared the performance of ChatGPT with that of medical students in a progress test.

**Methods:**

A total of 400 multiple-choice questions (MCQs) from the progress test in German-speaking countries were entered into ChatGPT’s user interface to obtain the percentage of correctly answered questions. We calculated the correlations of the correctness of ChatGPT responses with behavior in terms of response time, word count, and difficulty of a progress test question.

**Results:**

Of the 395 responses evaluated, 65.5% of the progress test questions answered by ChatGPT were correct. On average, ChatGPT required 22.8 s (SD 17.5) for a complete response, containing 36.2 (SD 28.1) words. There was no correlation between the time used and word count with the accuracy of the ChatGPT response (correlation coefficient for time rho = −0.08, 95% CI [−0.18, 0.02], t(393) = −1.55, *p* = 0.121; for word count rho = −0.03, 95% CI [−0.13, 0.07], t(393) = −0.54, *p* = 0.592). There was a significant correlation between the difficulty index of the MCQs and the accuracy of the ChatGPT response (correlation coefficient for difficulty: rho = 0.16, 95% CI [0.06, 0.25], t(393) = 3.19, *p* = 0.002).

**Conclusion:**

ChatGPT was able to correctly answer two-thirds of all MCQs at the German state licensing exam level in Progress Test Medicine and outperformed almost all medical students in years 1–3. The ChatGPT answers can be compared with the performance of medical students in the second half of their studies.

## Introduction

The use of artificial intelligence (AI) to assist with decision-making is becoming common, and its use in medical education is increasing. ChatGPT is an AI with a natural language processing (NLP) model (GPT-3.5) that can generate human-like responses to user input. It uses deep-learning algorithms that have been programmed for very large datasets and can be used in a wide variety of fields. However, its suitability for medicine has not been clarified. In this study, we evaluated the quality of AI’s responses by determining the correctness of the responses it provided in medical-related multiple-choice questions.

Teaching and testing medical knowledge is a central task in medical schools, as knowledge acquisition is a basic determinant of physicians’ performance [1,2]. Undergraduate medical education in Germany is designed as a six-year program, with the first five years primarily devoted to knowledge acquisition.

In Germany, a national competency-based catalog of learning objectives for undergraduate medical education (NKLM) was initiated in 2015 [3]. Most competencies described in the NKLM cover the acquisition of basic practical skills; however, in line with international practice [4], most of the overall curriculum is still based on teaching knowledge-based content.

At the same time, medical knowledge is advancing rapidly [5], and medical students must study harder to meet up with the knowledge required for success. The exponential growth of knowledge is a challenge for its users, especially in medicine. Moreover, the way physicians deal with knowledge resources available to them, such as literature search, greatly affects their success in the profession in terms of patients’ safety, quality assurance, among others. Medical students are required to learn these knowledge processing techniques at an early stage to integrate them into their academic work and later into their clinical practice. This is relevant to the extent that medical knowledge forms the basis for medical decisions that must be made, some of which can have serious consequences.

To assess cumulative increase in medical knowledge, progress testing is a globally popular tool, reliable tool for assessing medical knowledge [6], and can therefore be used to measure the increase in such knowledge. In German-speaking countries, medical schools are offered a progress test from the Berlin Charité, which 17 medical schools from Germany, Austria, and Switzerland have adopted. The following key elements of progress testing are described in a guide published by the Association for Medical Education in Europe (AMEE) [7].

- Administration to all students in an academic program
- Testing at regular intervals throughout the academic program
- Sampling complete knowledge domain expected of students at the end of their course, regardless of the student’s academic year.

Consequently, progress tests are comprehensive examinations of the complete final objectives of the curriculum [8]. As it is not summative, medical students typically do not prepare for the test. Students are discouraged from making blind guesses in a progress test through the option of ‘I do not know the answer’.

Moreover, as progress testing can be used to compare curricular changes [9–11], most faculties use it to monitor their students’ learning outcomes [e.g [12]. Generally, the German progress test shows a significant correlation with the German National Licensing Examination (criterion validity) [13]. Researchers have examined the generalizability of progress tests to larger contexts, such as the licensing examination. Scores on later progress tests were highly correlated with Step-1 performance [14,15], but there is also a relationship between growth trajectories obtained from progress tests and national licensing exams. Higher initial achievement levels and steepness of growth are positively related to performance in national licensing exams [16,17].

Therefore, medical students take progress tests in preparation for the licensing exam, and these multiple-choice exams can be taken after two as well as five years of study. Participation is mandatory for students from the 1st through 5th years of study, but may also be taken voluntarily in the 6th year of study. These exams are further supplemented by oral and practical assessments at various times, particularly in the final exams. The licensing exam requires approximately 60% of all multiple-choice questions (MCQs) to be answered correctly, although this threshold was lowered by a few percentage points after a national review process. Regarding assessment formats, research shows that variations in response formats, such as multiple-choice and constructed response, have little effect on actual assessment outcomes, with high correlations typically found between performance on tests using both formats [18,19]. MCQs can be constructed to assess higher order skills, including clinical reasoning tasks [20–23].

ChatGPT, as an AI language model, primarily has access to information rather than deep knowledge. Defining knowledge is a challenge, with several definitions proposed. Anderson et al.’s knowledge dimensions, part of the revised Bloom’s Taxonomy, assist teachers in planning and assessing learning activities [24]. The original taxonomy, developed in 1956 by Benjamin Bloom and colleagues [25], was revised in 2001 to include the Knowledge Dimension and the Cognitive Process Dimension, which classify the type of knowledge to be learned and describe cognitive processes involved in learning, respectively, to better reflect the contemporary understanding of the cognitive domain. In their Taxonomy Table, Anderson et al. [24] identified four categories of knowledge: Factual, Conceptual, Procedural, and Metacognitive Knowledge. Factual Knowledge consists of basic elements necessary for understanding a discipline or solving problems. Factual knowledge is subdivided into knowledge of terminology (specific facts and details) and knowledge of specific details and elements (basic components of a subject). In contrast to Factual Knowledge, Conceptual Knowledge”[…] is knowing the interrelationships among the basic elements within a larger structure that enable them (the elements) to function together.” [26]. Given the nature of ChatGPT’s responses to a wide array of questions, ChatGPT’s answers frequently seem to exhibit at least factual knowledge, as it is capable of providing specific details, terminology, and elements within various subject areas.

### Problem statement

Dialog-based interaction with ChatGPT makes this information resource an attractive alternative to other factual knowledge resources in the field of medicine that are primarily distributive and non-interactive. In particular, it is difficult to find an answer to a specific question in a textbook or internet database. In medical schools, techniques such as literature searches or decision paths must be learned to find an answer to what can be very complex medical questions. Conveniently, ChatGPT is available 24 h per day through an extremely simple input field, even on mobile devices, and provides a precise answer text instantly (without requiring thousands of hits). Therefore, AI enables interactive access to factual knowledge regardless of time or location, and medical students (and patients) are expected to use the service it provides for medical decisions in the future. It is also essential to evaluate the quality of medical decisions that ChatGPT provides.

### Research questions

To elicit the benefits of ChatGPT for medical education from a learner-centered perspective, we aimed to measure the performance of ChatGPT as a fictitious participant in Progress Test Medicine. Thus, this study answers the following research questions.

1. What is the percentage of correctly answered questions by the ChatGPT in Progress Test Medicine?
2. Is there any evidence of the strengths or weaknesses of the ChatGPT in specific medical specialties or organ systems?
3. Is the correctness of ChatGPT’s responses related to behavior in terms of response time, word count, and difficulty of a Progress Test question?
4. What is ChatGPT’s performance in Progress Test Medicine compared with that of medical students in different study years?

## Methods

To adopt a learner-centered perspective, we designed the data collection by mimicking the expected behavior of medical students when asked to answer MCQs. It takes six years to complete a course in medical school in Germany, with students enrolled directly from secondary schools. The course of study is divided into a pre-clinical section (the first two years) and a clinical section (the last four years). To improve students’ clinical experience, they are rotated in various hospital departments during their final year (‘clinical/practical’ year).

Instead of using a system interface (application programming interface or API, also offered in the future for the chatbot), ChatGPT was accessed with a mobile device via the publicly offered user interface at chat.openai.com. To do this, we created an account via an e-mail address and confirmed a code sent thereafter on a smartphone. After logging into the website, a single-line input field is available for communication with the chatbot.

### Study design

The principal researchers collected all questions of the Progress Test Medicine in the 2021–2022 academic year and entered them into the ChatGPT interface (latest version dated 9 January 2023). Each Berlin Progress Test consists of 200 MCQs offered biannually, or a total of 400 questions. There is a single best answer for each question. The MCQs were selected from a database of items and matched to a blueprint. Once included in the test, the questions were not used for two years to prevent items from being collected and easily retrieved [27]. Students were asked to take the test within a time frame of a maximum of three hours. The MCQs were distributed across 27 medical specialties and 14 organ systems, listed in Table 1.

**Table 1.** Distribution of MC questions among specialties and organ systems. Frequencies are given in absolute numbers and %.

Distribution of MC-Questions among specialties and organ systems	N	Overall, *N* = 400^*1*^	Summer term 2022, *N* = 200^*1*^	Winter term 2021–2022, *N* = 200^*1*^
**Specialty**	400			
Anatomy, Biology		23 (5.8%)	12 (6.0%)	11 (5.5%)
Anesthesiology, Emergency Medicine and Intensive Care		18 (4.5%)	9 (4.5%)	9 (4.5%)
Biochemistry, Chemistry, Molecular Biology		16 (4.0%)	5 (2.5%)	11 (5.5%)
Clinical Chemistry, Clinical Pathology		3 (0.8%)	2 (1.0%)	1 (0.5%)
Dermatology		7 (1.8%)	4 (2.0%)	3 (1.5%)
Epidemiology, Medical Biometrics		11 (2.8%)	4 (2.0%)	7 (3.5%)
General Practice		29 (7.2%)	11 (5.5%)	18 (9.0%)
Gynecology and Obstetrics		18 (4.5%)	11 (5.5%)	7 (3.5%)
Human Genetics		8 (2.0%)	4 (2.0%)	4 (2.0%)
Hygiene, Microbiology		11 (2.8%)	5 (2.5%)	6 (3.0%)
Internal Medicine		67 (17%)	32 (16%)	35 (18%)
Legal Medicine		8 (2.0%)	3 (1.5%)	5 (2.5%)
Med. Psychology/Sociology		9 (2.2%)	5 (2.5%)	4 (2.0%)
Naturopathy, Physical Medicine		1 (0.3%)	0 (0%)	1 (0.5%)
Neurology		21 (5.2%)	11 (5.5%)	10 (5.0%)
Occupational and Social Medicine, Healthcare		8 (2.0%)	4 (2.0%)	4 (2.0%)
Ophthalmology		7 (1.8%)	4 (2.0%)	3 (1.5%)
Orthopedics		9 (2.2%)	5 (2.5%)	4 (2.0%)
Otorhinolaryngology		7 (1.8%)	5 (2.5%)	2 (1.0%)
Pediatrics		21 (5.2%)	12 (6.0%)	9 (4.5%)
Pathology		12 (3.0%)	6 (3.0%)	6 (3.0%)
Pharmacology, Toxicology		23 (5.8%)	11 (5.5%)	12 (6.0%)
Physiology, Physics		16 (4.0%)	7 (3.5%)	9 (4.5%)
Psychiatry, Psychosomatic Medicine		20 (5.0%)	11 (5.5%)	9 (4.5%)
Radiology, Nuclear Medicine		5 (1.3%)	4 (2.0%)	1 (0.5%)
Surgery		15 (3.8%)	9 (4.5%)	6 (3.0%)
Urology		7 (1.8%)	4 (2.0%)	3 (1.5%)
**Organ system**	400			
Blood, immune system		26 (6.5%)	13 (6.5%)	13 (6.5%)
Cardiac system		44 (11%)	22 (11%)	22 (11%)
Cell		22 (5.5%)	11 (5.5%)	11 (5.5%)
Digestive system		36 (9.0%)	18 (9.0%)	18 (9.0%)
General medicine		20 (5.0%)	10 (5.0%)	10 (5.0%)
Hormones, metabolism		26 (6.5%)	13 (6.5%)	13 (6.5%)
Methods		14 (3.5%)	7 (3.5%)	7 (3.5%)
Musculoskeletal system		30 (7.5%)	15 (7.5%)	15 (7.5%)
Neurosystem, brain, senses		32 (8.0%)	16 (8.0%)	16 (8.0%)
Psychosocial system		40 (10%)	20 (10%)	20 (10%)
Reproductive system		22 (5.5%)	11 (5.5%)	11 (5.5%)
Respiratory system		44 (11%)	22 (11%)	22 (11%)
Skin		18 (4.5%)	9 (4.5%)	9 (4.5%)
Urinary system		26 (6.5%)	13 (6.5%)	13 (6.5%)
^*1*^Frequency (in %)				

Data collection for this study was determined à priori as follows:

- We submitted the full MCQ via the single-line input window using copy-and-paste. The MCQs were entered including case vignette, question wording, and all answer options (including the ‘don’t know’ option).
- The time measurement for answering the question by the ChatGPT started with pressing the Enter button. In the answer line of ChatGPT, a cursor blinks during the processing and answering of a question.
- The answer provided by ChatGPT in the communication field of the user interface became inactive until no more text characters were added, and the blinking of the cursor stopped. We did not use the option to stop generating ChatGPT’s response, which was introduced with the ChatGPT-release of Jan 9, 2023. Then, the answer was copied into a file, from which the next question was exported back to ChatGPT via copy and paste.
- The Enter button for the next question is activated once the previous question is answered and the time measurement of answering the question stops.

The copy and paste took 10 s. The remaining time was recorded to obtain the response time for each question.

Due to high public interest in AI, morning time slots were chosen for chatbot interactions to avoid busy periods with U.S. users. This helped to mitigate any artificial delay in response times caused by the limited computational capabilities of the version used.

To closely represent the student’s perspective, technical optimizations to AI access were not employed. The study used a generally available user interface instead of an API and input questions without additional formatting to avoid increasing the readability of the AI. Questions were not translated from German to English, as this could have affected student comprehension due to language barriers. Moreover, instructions such as ‘Please select only from the given answers’ or ‘Please choose only one of the given answers’ were not used, and no feedback on answer correctness was provided to ChatGPT, as AI learns and improves from such feedback.

After entering and answering all MCQs, the account used and data available in the tool were completely deleted in accordance with the procedure specified by the company (OpenAI, L.L.C., San Francisco, CA).

### Outcome measure

As relevant outcome measures, the solution given in ChatGPT’s answer was assigned according to possible answers to the MC question. For this, the exact wording of the answer to the MC question had to be reproduced in the answer to the ChatGPT. Matches in the Progress Test question were classified as correct and the assigned answers from ChatGPT were counted as correct answers, and all other answers as incorrect.

Responses that were not interpretable or were multiple or alternative responses, of which one or more were correct, were valued as ‘NA’ (not applicable). The timing described above was measured in seconds per answer using the tool. The answers were registered in terms of volume as respective word counts.

### Statistical methods

The proportion of correct responses, response time, and word volumes are described and correlated. Furthermore, the point biserial correlation of the respective answers was calculated using the difficulty index.

We reported the number of individual students per year of study and counted the number of questions that were answered correctly. The distribution of the percentage of correctly answered questions is shown per study year, including the mean and standard deviation. Given the anonymity of the test data and general data protection, medical student cohorts cannot be described by any socio-demographic factors. The results of ChatGPT were compared with those of the students from the respective years regarding the overall result using one-sided one sample z-tests for proportions. In addition, we wanted to show the relationship between the percentage of correct answers (test score) per medical specialty and organ system using radar charts.

Statistical analysis was conducted and tables and figures were created using R [28] in RStudio IDE (Posit Software, Boston, MA) with the tidyverse, gt and ggradar packages [29–31].

## Results

**Figure 1.**
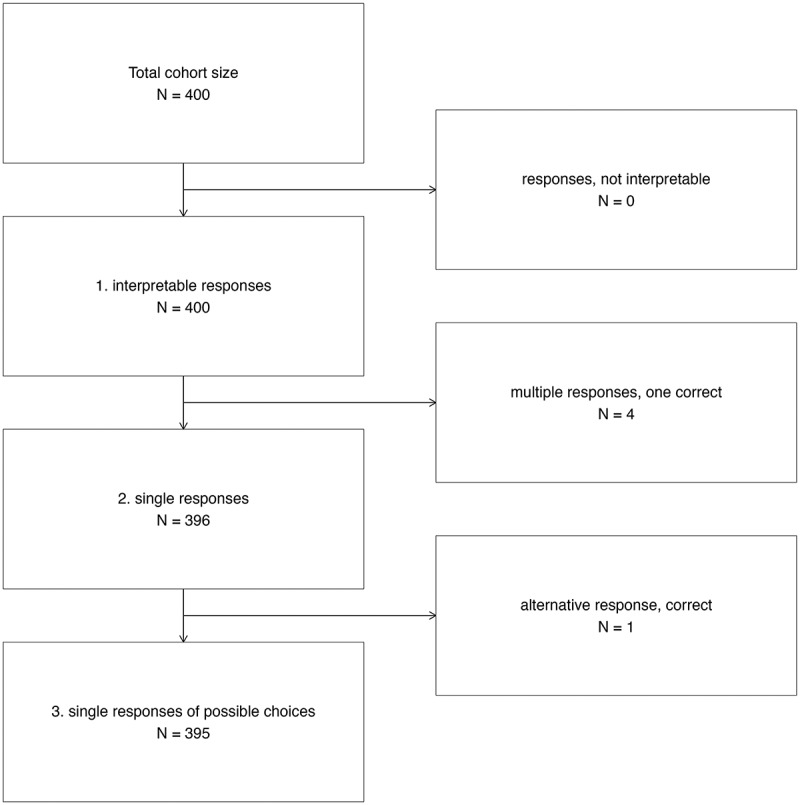
Flowchart of evaluable responses from ChatGPT to MC questions from Progress Test Medicine.

A total of 400 MCQs were entered into the tool, of which 395 could be evaluated. The reasons for excluding irregular answer patterns are shown in Figure 1. The percentage of answers identical to the wording of the given multiple-choice options was 99.0%. These answers were often further elaborated by explanatory text, and 71.5% of them additionally offered the identical given alphabetical listing format (e.g., ‘a) … ’) of the MCQ options.

**Figure 2.**
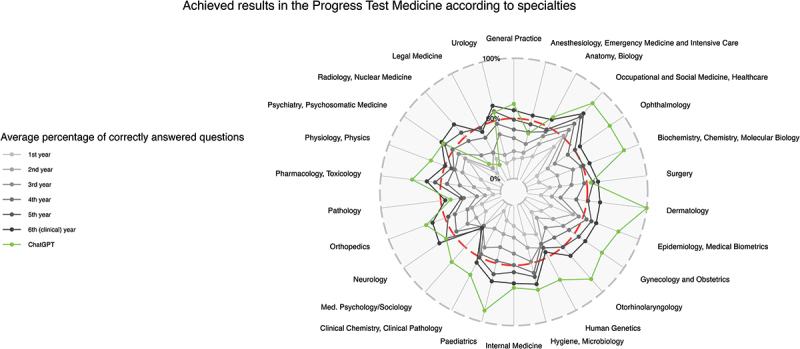
Results achieved in the progress test medicine according to specialties. Radar chart with achieved results as mean of correct answers in %; the pass mark for the state exam is plotted as a red dashed line.

**Figure 3.**
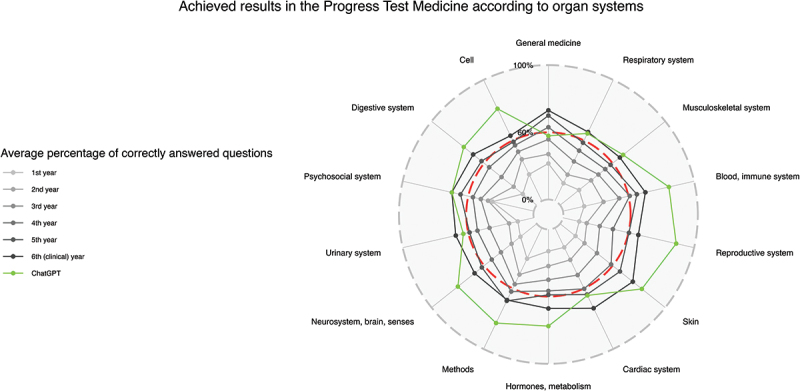
Results achieved in the progress test medicine according to organ systems. Radar chart with achieved results as mean of correct answers in %; the pass mark for the state exam is plotted as a red dashed line.

After the evaluation of all data sets, the following main results were obtained. In total, 65.5% of the progress test questions answered by ChatGPT were correct (see Table 2). The percentages of correct questions compared to the average of all students regarding different medical specialties and organ systems are presented in Figures 2 and 3, respectively. ChatGPT shows low mean scores in legal medicine (14.3%, SD 37.8%) and radiology (20.0%, SD 44.7%) and a high mean score in dermatology (100.0%, SD 0.0%), but with non-significant z-values in comparison to ChatGPT’s overall score (−1.08, −0.96 and 0.73, respectively).

**Table 2.** Progress testing results: Medical students vs ChatGPT.

Participants	Number of single participations	Study progress	Correct Answers (mean %)	SD (%)	z-score	p-value
Medical students	3,390	1^st^ year	18.01	11.90	3.99	p<.001 *
Medical students	4,783	2^nd^ year	26.88	13.11	2.95	p=.002 *
Medical students	3,689	3^rd^ year	37.05	15.39	1.85	p=.032 *
Medical students	3,125	4^th^ year	45.90	17.83	1.10	p=.136
Medical students	3,390	5^th^ year	52.73	17.91	0.71	p=.238
Medical students	467	6^th^ (clinical) year	60.69	18.76	0.26	p=.399
ChatGPT	2	GPT-3.5	65.5	47.3		

Note: * = significant with respect to *p* ≤ .05 level.

Source: Berlin Progress Tests results; Study year 2021/2022.

On average, ChatGPT required 22.8 seconds (SD 17.5 seconds, median 19.6 seconds) for a complete response according to our time measurement method. Each answer given by ChatGPT contained, on average, over 35 words (36.2 ± 28.1 words). Among these, the shortest responses consisted of two words, and the longest, 144 words. There was no correlation between time used and word count with the accuracy of the ChatGPT response (correlation coefficient for time rho = −0.08, 95% CI [−0.18, 0.02], t(393) = −1.55, *p* = 0.121; for word count rho = −0.03, 95% CI [−0.13, 0.07], t(393) = −0.54, *p* = 0.592).

However, there was a significant correlation between the difficulty index of the questions and the accuracy of the ChatGPT response (correlation coefficient for difficulty rho = 0.16, 95% CI [0.06, 0.25], t(393) = 3.19, *p* = 0.002), meaning that the easier questions (for medical students) were more often answered correctly.

## Discussion

We compared the performance of ChatGPT with that of the medical students in a Progress Test Medicine to assess the former’s strengths and weaknesses in specific medical specialties or organ systems. We examined whether the correctness of ChatGPT’s responses was related to behavior in terms of response time, word count, and difficulty of a Progress Test question and compared ChatGPT’s performance with that of medical students in different study years.

Here, we demonstrate the potential power of large-language models in medicine. ChatGPT was able to correctly answer two-thirds of all questions at the German state exam level in Progress Test Medicine, indicating that it outperformed almost all medical students in years 1–3. Only students from the 4th year onwards achieved similar results but did not still outperform. There were no indications of the strengths or weaknesses of ChatGPT in specific medical specialties or organ systems. ChatGPT can answer easier MCQs better than difficult ones, but we did not find a correlation between the response time and response length (in words) and correctness.

### Primary and secondary outcomes

From the students’ perspective, the central question regarding the use of a learning medium is whether it helps them in their medical education. In other words, does this application help students to learn? In applying the tool, it was fascinating to observe the speed at which even complex case vignettes were processed. For almost all queries, AI immediately starts to answer the question and produces a linguistically high-quality, well-structured, and logical text that is very easy to follow. Unfortunately, these results apply to all answers given by ChatGPT; therefore, it is not possible to determine whether the answer is correct or incorrect. It is also counterintuitive that neither the time it takes ChatGPT to provide an answer nor the length of the answer is related to the correctness. Normally, one would expect that a counterpart with a quick or particularly detailed answer would have a greater tendency to be correct. To increase medical knowledge, it is essential to ensure that the factual knowledge learned is at the current correct level that research can offer. Otherwise, students run the risk of learning incorrect information, which is not desirable. Therefore, the uncertainty about the answers from ChatGPT limits its usefulness for medical education in this regard.

With the results obtained in this study, the answers were correct in two out of three cases, and the confidence in the solution offered by the ChatGPT increased. One can infer, perhaps, that the strength of AI lies in challenging (presumed) medical knowledge. Thus, in their acquisition of knowledge, medical students as well as doctors on the ward could compare their suspected diagnoses or therapy suggestions with those of the AI in order to prevent medical decision errors. For this, however, AI answers would have to be better or even perfect. The same applies to the (qualitative) review process of exam questions for which ChatGPT is ideally suited. By entering the questions, they can be quickly and cheaply examined for ambiguities in the wording. Additionally, due to the extensive feedback provided by AI, hints of unwanted clues can sometimes be found in the answer options.

Answering MCQ via AI can be both helpful and problematic, depending on the context. When used as a tool to help students understand the material, AI can provide valuable guidance by giving information. However, if students rely solely on AI for answers without engaging in critical thinking or problem-solving, it may hinder their learning. If students even use it to obtain answers dishonestly, it can certainly undermine the assessment of their knowledge. Ideally, AI should be used as a tool to support students in their learning process, helping them deduce the answer through a series of hints or guiding questions. This approach aligns with the medical thinking and problem-solving skills that are crucial in a professional context [32].

Notably, due to the nature of the algorithm, ChatGPT does not seem to be able to express uncertainty. For example, the AI did not answer ‘don’t know’ a single time, even though this answer option was also available in every question asked. Wrong answers are just as convincingly justified as correct ones, a behavior that is not uncommon in large language models and is sometimes referred to as ‘hallucination.’ Dealing with and expressing uncertainty is an integral part of scientific education. Unfortunately, the associated risk literacy, that is, the ability to correctly assess and understand information about risk [33], among medical professionals, while slightly above average compared to the general population, is also not particularly high in absolute terms [34]. Moreover, there are indications that risk literacy does not improve with medical education and training [34,35]. However, it is a prerequisite for effective risk communication [36–38]], and is thus essential for informed medical decision-making by both physicians [39] and patients [40].

Future research on the content analysis of ChatGPT responses is necessary. For example, we felt that ChatGPT had problems in the differential diagnosis of chest pain. ChatGPT is also expected to improve through (also announced) updates. A potential follow-up study could use a similar design to compare the performance of the updated models with our results and see to what extent the AI learns, i.e., shows progress in medical factual knowledge.

### Limitations

A possible influence of the study framework on the interpretation and applicability of the results is the selection of the progress test questions. For example, some medical specialties, such as legal medicine, orthopedics, and otorhinolaryngology, are tested with very few questions, which severely limits the generalizability of the results for individual medical specialties. Progress test questions also map only a portion of the skills and abilities necessary for professional medicine. It is essential to recognize that ChatGPT is incapable of replicating the full range of skills and abilities that medical professionals possess.

The study design was dominated by the ‘everyday’ approach to AI, which was not designed to show the maximum performance of the model. Thus, the validity of technical response behavior is limited. The response time of ChatGPT depends on a combination of internet speed, device performance, and server-side processing capabilities, and may vary depending on these factors. Thus, we chose a method that most closely illuminates the usability of AI for medical students.

## Conclusions

ChatGPT’s performance in answering medical questions demonstrates the potential of large language models. It outperformed almost all German medical students in years 1–3 in the Progress Test Medicine, but we found no indications of its strengths or weaknesses in specific medical specialties or organ systems. ChatGPT can answer easier MCQs better than difficult ones, but there is no correlation between the response time and length (in words) with correctness.

Medical students (and physicians) should understand the strengths and weaknesses of these tools to maximize their impact on diagnosis and therapy. It will be the task of medical educators to positively guide this process whenever they are applying them. However, it’s essential to use such AI-driven models responsibly and ethically in academic settings, after considering the potential limitations and the importance of fostering critical thinking in students.
